# A Systematic Review and Meta-Analysis Comparing Programmed Intermittent Bolus and Continuous Infusion as the Background Infusion for Parturient-Controlled Epidural Analgesia

**DOI:** 10.1038/s41598-019-39248-5

**Published:** 2019-02-22

**Authors:** Jiqian Xu, Jie Zhou, Hairong Xiao, Shangwen Pan, Jie Liu, You Shang, Shanglong Yao

**Affiliations:** 1 Department of Anesthesiology, Union Hospital, Tongji Medical College, Huazhong University of Science and Technology, Wuhan, 430022 China; 2 Department of Anesthesiology, North Sichuan Medical College Affiliated Hospital, Nanchong, 637000 China; 3 Institute of Anesthesiology and Critical Care Medicine, Union Hospital, Tongji Medical College, Huazhong University of Science and Technology, Wuhan, 430022 China; 4 Red Cross central blood station of Nanchong, Sichuan, Nanchong 637000 China

## Abstract

The programmed intermittent epidural bolus (PIEB) technique offers multiple benefits over continuous epidural infusion (CEI), but controversy still exists when it is used in conjunction with a parturient-controlled epidural analgesia (PCEA) regimen. A systematic review and meta-analysis was thus conducted using the Medline, EMBASE, CENTRAL and Web of Science databases with the aim of identifying those randomized controlled trials (RCTs) that performed a comparison between PIEB and CEI in healthy parturients using a PCEA regimen with regard to the duration of labor, labor pain, anesthesia interventions, maternal satisfaction and main side effects. The data were analyzed using a random-effects model. Eleven eligible trials were included, in which 717 participants were allocated to the PIEB + PCEA group and 650 patients were allocated to the CEI + PCEA group. The rate of instrumental delivery, incidence of breakthrough pain, PCEA usage rates and local anesthetic usage were significantly reduced, the labor duration was statistically shorter, and the maternal satisfaction score was significantly improved in the PIEB + PCEA group compared with that in the CEI + PCEA group. There were no differences in the side effects between the two groups. The results of the present study suggest that the PIEB technique in conjunction with the PCEA regimen was more advantageous than CEI + PCEA, but additional studies should be conducted to consistently demonstrate an improvement in the maternal and fetal obstetric outcomes.

## Introduction

Labor pain is one of the most painful experiences for a woman^1^. The degree and relief of pain affects maternofetal physiology and neuropsychology, as well as maternal satisfaction^2^. It is thus necessary that feasible analgesia is used to improve maternal satisfaction and decrease the side effects on the mother and fetus^3^. It has been acknowledged that patient-controlled epidural analgesia (PCEA) is an effective method of labor analgesia that has been associated with superior maternal satisfaction and a lower incidence of adverse events compared with other analgesia techniques^4,5^; however, the PCEA regimen without a background infusion is not beneficial for decreasing pain scores of the parturient and the workload of the medical staff. Therefore, the continuous epidural infusion (CEI) technique added to the PCEA regimen has become a standard labor epidural analgesic regimen in North America and Europe in recent decades^5–7^, but CEI + PCEA could increase the risk of instrumental delivery and prolong the second stage of labor compared with PCEA-only epidural labor analgesia^8^, and it is controversial whether there is a decrease in local anesthetic (LA) usage and an improvement in analgesic efficacy with CEI + PCEA compared to PCEA alone^6,8^.

Compared with CEI + PCEA, the computer-integrated PCEA regimen comprising a PCEA regimen plus a programmed intermittent epidural bolus (PIEB), which automatically administers epidural solution at scheduled intervals, has resulted in a lower incidence of motor block and instrumental delivery^9^, lower rate of requiring rescue boluses^10^, and greater patient satisfaction^10,11^. However, it has been demonstrated that the PIEB + PCEA mode has similar LA consumption, motor blockade, instrumental delivery and breakthrough pain rates compared with the PCEA or CEI + PCEA regimens^5,9,12–14^. Therefore, the aim of this systemic review and meta-analysis was to compare whether PIEB in conjunction with PCEA in healthy pregnancy improved delivery mode, labor analgesia, patient satisfaction, maternal and neonatal obstetric outcomes compared to the CEI + PCEA regimens.

## Methods

### Approval

Our IRB did not require ethics approval because there were no data directly collected from patients. We evaluated and synthesized only data in published trials.

### Search Strategy

The study was performed and reported in accordance with the recommended methods of the PRISMA checklist (Supplementary Appendix S1). The Medline, EMBASE, CENTRAL and Web of Science databases were searched by two authors independently without filters and language restrictions. Available articles were updated through February 15, 2018. The MeSH terms “epidural analgesia” or “pregnancy” and text words “intermittent”, “continuous”, “automated” and “programmed” as well as relevant synonyms were searched and then these results were combined. The exhaustive search strategy is described in the supplemental material (Supplemental Appendix S2). The references of the retrieved articles and relevant reviews were screened to identify additional studies. We attempted to contact the authors when the original data were missing. We did not search for unpublished data or trials.

### Study Selection and Quality Assessment

The potentially eligible trials were independently examined at the title/abstract level by two investigators. The divergences between the 2 authors were settled by consensus and discussion with a third author. The studies considered for this analysis included any published RCTs comparing the PIEB + PCEA regimen and the CEI + PCEA regimen in healthy parturients. These subjects received lumbar epidural catheters for maintaining labor analgesia. The studies that did not clearly describe the methods of delivering the PIEB, CEI and PCEA and the protocols for maintaining labor analgesia were excluded. Abstracts of scientific meetings, termination of pregnancy, and trials in which PCEA was not available in both the PIEB and CEI groups were also excluded.

The risk of bias of all eligible trials was evaluated by two independent investigators based on “Risk of Bias Assessment Tool” adapted by the Cochrane Back and Neck (CBN) Group^15^. Each of the 12 criteria was scored as yes, no, or unsure, and if at least 6 of the 12 criteria were scored as “yes”, the trial was rated as having a “low risk of bias”^16^. Any divergences that occurred were settled by consulting a third author.

### Data Extraction and Management

The primary outcomes were the evaluation of the mode of delivery (instrumental vaginal or cesarean delivery, CD), duration of labor, efficacy of analgesia and anesthesia interventions, which was reflected by visual or verbal analog scale (VAS, 0–100 mm) scores, incidence of breakthrough pain, PCEA usage rate, time to first PCEA or breakthrough pain, LA dose delivered per hour, and patient satisfaction. The secondary outcomes included the degree of sensory blockade and motor blockade, the incidence of pruritus, hypotension, nausea and vomiting. Apgar scores were determined at 1 minute and 5 minutes. Studies were included if they reported any of the primary outcomes. Two investigators independently extracted the relevant data. All data were tabulated in Microsoft Excel. Any discrepancies regarding the inclusion of eligible trials were handled by discussion; if a consensus was not achieved, the opinion of the third investigator was sought. If the data were reported as medians, ranges, and confidence intervals (CIs), the mean and standard deviations were calculated as previously described^17^. The epidural LA dose per hour from the summarized data and milligram equivalents of ropivacaine per hour of anesthesia delivery were calculated as previously described^16^; ropivacaine and levobupivacaine were assumed to be approximately 60% as potent as bupivacaine^18–20^.

### Statistical Analysis

Pooled analysis was conducted with Review Manager Version 5.3 (Cochrane, London, UK). An inverse variance random-effects model was applied due to clinical or methodological heterogeneity across studies. Continuous parameters were weighted using the mean difference (MD) with 95% CI, and binary variables were calculated using the odds ratios (OR) with 95% CI. The *I*^2^ statistic was used to assess the heterogeneity of the trials. *P* values < 0.1 were predefined as indicating statistically significant heterogeneity. *I*^2^ > 50% was viewed as indicating heterogeneity. It was decided a priori that the primary outcome would be subanalyzed according to the technique (subarachnoid or epidural anesthesia) used to initiate labor analgesia, if possible. Sensitivity analyses were performed using the leave-one-out method to explore the heterogeneity; if particular studies were methodologically different from the others, these studies were excluded. When meta-analytic methods were unavailable due to different types of data or significant heterogeneity in the outcome effect, a qualitative descriptive analysis was considered for these data. Publication bias was appraised with the Egger test using Stata version 12.0, and *P* < 0.05 indicated a significant publication bias.

## Results

### Characteristics of the Included Studies

**Figure 1:**
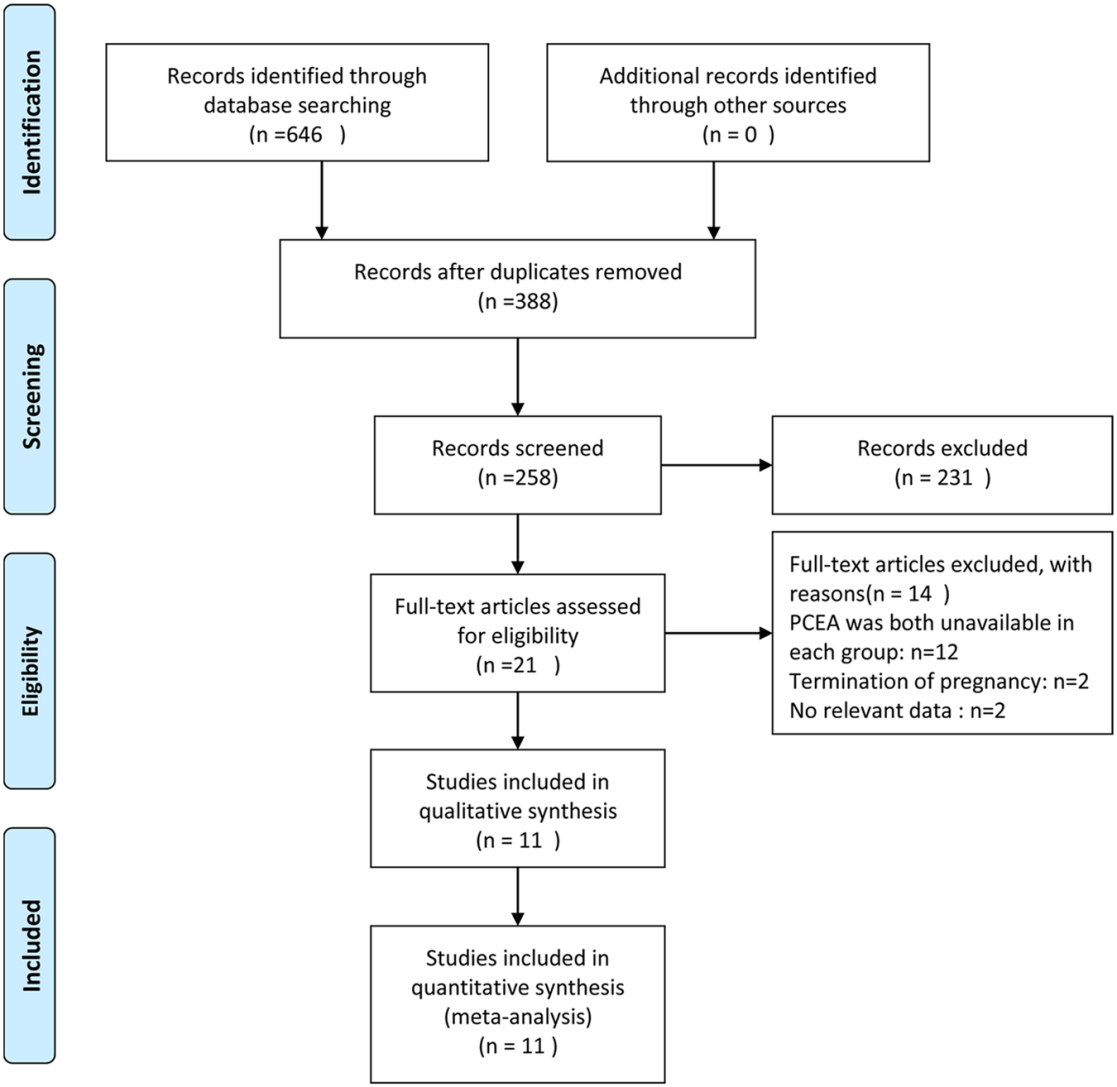
Study selection flow diagram.

**Figure 2:**
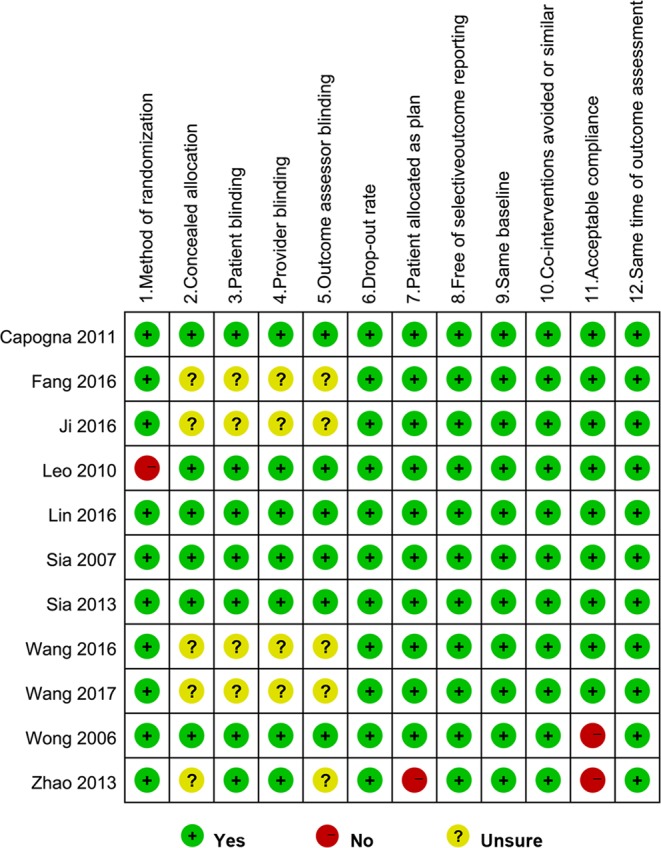
The risk of bias for the included studies.

A total of 646 records were initially searched, and 388 records were removed as duplicates. A total of 231 irrelevant records were excluded, and 27 articles remained for in-depth, full text review after screening titles or abstracts. Finally, eleven potentially eligible articles were retrieved for this systematic review^9–11,21–27^. The flow diagram is depicted in Fig. 1. The eleven trials included 1367 participants, of whom 717 subjects used the PIEB + PCEA regimen and 650 patients applied the CEI + PCEA regimen for maintaining labor analgesia in the study, and the characteristics of the individual studies are summarized in Table 1. Five studies initiated labor analgesia with a combined spinal-epidural (CSE) technique^10,11,24,25,27^. The risk of bias is shown in Fig. 2, and all 11 trials were considered to be at low risk of bias (Table 1).

**Table 1:** Study Characteristics and Risk of Bias Assessment of the Included Studies.

Study, year	Country	*N*	Parity	Neuraxial Analgesia Initiation (medications)	Epidural maintenance solution (drug/concentration)	Maintenance of analgesia + treat breakthrough pain		Risk of bias assessment
						PIEB + PCEA regimens *(n)*	CEI + PCEA regimens *(n)*	
Capogna *et al*.^9^	Italy	145	Nulliparous	**EA** (levobupivacaine, 0.0625%; sufentanil, 10 µg/mL; 20 mL)	Levobupivacaine, 0.0625%- 0.125%; sufentanil, 0.5 µg/mL	10 mL (0.0625%) bolus every hour + PCEA (5 mL bolus, 10 minute lockout, 0.125%) (***n*** = ***75***)	10 mL/h (0.0625%) + PCEA (5 mL bolus, 10 minute lockout, 0.125%) (***n*** = ***70***)	Low risk
Fang *et al*.^21^	China	200	Nulliparous	**EA** (ropivacaine, 0.075%; sufentanil, 0.5 µg/mL; 10 mL)	Ropivacaine, 0.075%; sufentanil, 0.5 µg/mL	8 mL (0.075%) bolus every hour + PCEA (6 mL bolus, 15 minute lockout, 0.075%) (***n*** = ***100***)	8 mL/h (0.075%) + PCEA (6 mL bolus, 15 minute lockout, 0.075%) (=***100***)	Low risk
Ji *et al*.^22^	China	50	Nulliparous	**EA** (ropivacaine, 0.075%; sufentanil, 0.3 µg/mL; 8 mL)	Ropivacaine, 0.075%; sufentanil, 0.3 µg/mL	8 mL (0.075%) bolus every hour (or 20 minutes after successful PCEA dose) + PCEA (5 mL bolus, 20 minute lockout, 0.075%) (***n*** = ***25***)	8 mL/h (0.075%) + PCEA (5 mL bolus, 20 minute lockout, 0.075%) (***n*** = ***25***)	Low risk
Leo *et al*.^10^	Singapore	62	Nulliparous	**SA** (ropivacaine, 2 mg; fentanyl, 15 µg)	Ropivacaine 0.1%; fentanyl, 2 µg/mL	5 mL (0.1%) bolus every hour (or 30 minutes after successful PCEA dose) + PCEA (5 mL bolus, 10 minute lockout, 0.1%) (***n*** = ***31***)	5 mL/h (0.1%) + PCEA (5 mL bolus, 10 minute lockout, 0.1%) (***n*** = ***31***)	Low risk
Lin *et al*.^23^	China	197	Nulliparous	**EA** (ropivacaine, 0.15%; 10 mL)	Ropivacaine, 0.1%; sufentanil, 0.3 µg/mL	5 mL (0.1%) bolus every hour + PCEA (5 mL bolus, 20 minute lockout, 0.1%) (n = 98)	5 mL/h (0.1%) + PCEA (5 mL bolus, 20 minute lockout, 0.1%) (***n*** = ***99***)	Low risk
Sia *et al*.^25^	Singapore	42	Nulliparous	**SA** (ropivacaine, 2 mg; fentanyl, 15 µg)	Ropivacaine 0.1%; fentanyl, 2 µg/mL	5 mL (0.1%) bolus every hour (or 1 hour after successful PCEA dose) + PCEA (5 mL bolus, 10 minute lockout, 0.1%) (***n*** = ***21***)	5 mL/h (0.1%) + PCEA (5 mL bolus, 10 minute lockout, 0.1%) (***n*** = ***21***)	Low risk
Sia *et al*.^24^	Singapore	102	Nulliparous	**SA** (ropivacaine, 2 mg; fentanyl, 15 µg)	Ropivacaine 0.1%; fentanyl, 2 µg/mL	5 mL (0.1%) bolus every hour (or different time after successful PCEA dose) + PCEA (5 mL bolus, 10 minute lockout, 0.1%) (***n*** = ***51***)	5 mL/h (0.1%) + PCEA (5 mL bolus, 10 minute lockout, 0.1%) (***n*** = ***51***)	Low risk
Wang *et al*.^26^	China	200	Nulliparous	**EA** (ropivacaine, 0.125%; sufentanil, 0.4 µg/mL 10 mL)	Ropivacaine 0.08%; sufentanil, 0.4 µg/mL	10 mL (0.08%) bolus every hour + PCEA (5 mL bolus, 30 minute lockout, 0.1%) (***n*** = ***100***)	10 mL/h (0.08%) + PCEA (5 mL bolus, 30 minute lockout, 0.1%) (***n*** = ***100***)	Low risk
Wang *et al*.^28^	China	186	Nulliparous	**EA** (ropivacaine, 0.125%; sufentanil, 0.4 µg/mL 10 mL)	Ropivacaine 0.08%; sufentanil, 0.4 µg/mL	10 mL (0.08%) bolus 0.5 or 1 hour + PCEA (5 mL bolus, 30 minute lockout, 0.1%) (***n*** = ***124***)	10 mL/h (0.08%) + PCEA (5 mL bolus, 30 minute lockout, 0.1%) (***n*** = ***62***)	Low risk
Wong *et al*.^11^	USA	126	Parous	**SA** (bupivacaine, 1.25 mg; fentanyl, 15 µg)	Bupivacaine, 0.625%; fentanyl 2, µg/mL	6 mL (0.625%) bolus every 30 minutes + PCEA (5 mL bolus, 10 minute lockout, 0.625%) (***n*** = ***63***)	12 mL/h (0.625%) + PCEA (5 mL bolus, 10 minute lockout, 0.625%) (***n*** = ***63***)	Low risk
Zhao *et al*.^27^	China	57	Parous	**SA** (ropivacaine, 3 mg)	Ropivacaine 0.1%; sufentanil, 0.5 µg/mL	3 mL (0.1%) bolus every hour + PCEA (3 mL bolus, 10 minute lockout, 0.1%) (***n*** = ***29***)	6 mL/h (0.1%) + PCEA (3 mL bolus, 10 minute lockout, 0.1%) (***n*** = ***28***)	Low risk

PIEB, programmed intermittent epidural boluses; CEI: continuous epidural infusion; PCEA: patient-controlled epidural analgesia; **SA:** subarachnoid anesthesia; **EA:** epidural anesthesia.

### Mode of Delivery

**Figure 3:**
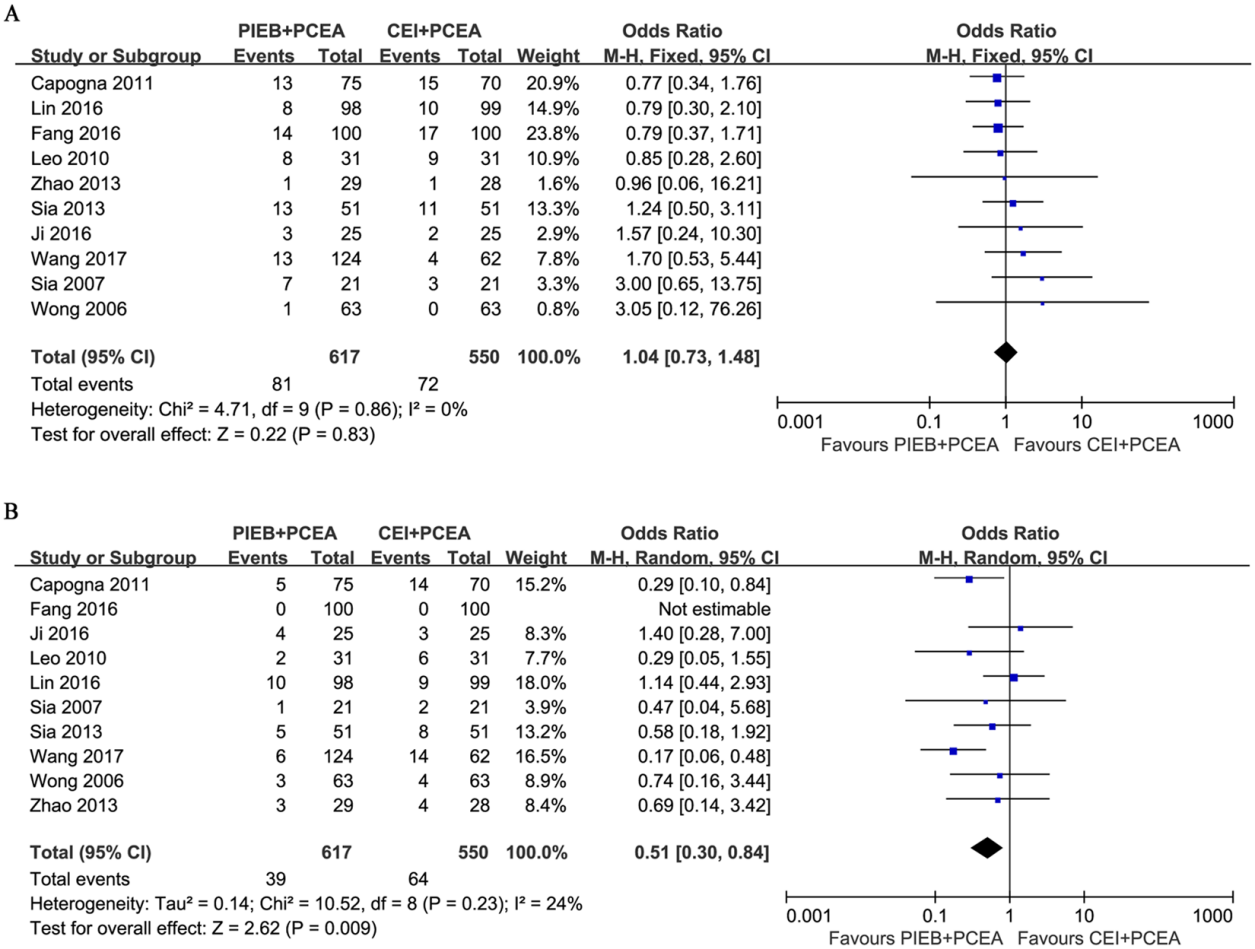
(**A**) Forest plot of the pooled analysis for mode of delivery (cesarean delivery). (**B**) Forest plot of the pooled analysis for mode of delivery (instrumental delivery). PIEB, programmed intermittent epidural boluses; CEI, continuous epidural infusion; PCEA: patient-controlled epidural analgesia; CI: confidence interval.

Ten trials, which included 1167 subjects, reported the mode of delivery. A significant difference in CD rate between the two groups was not present in these studies. The pooled analysis did not find a difference in the rate of CD (OR, 1.03; 95% CI, 0.72–1.47; Fig. 3A). Nine trials reported the number of instrumental deliveries, and the overall results showed a significant difference between the two groups (OR, 0.51; 95% CI, 0.30–0.84; Fig. 3B). Eight trials, which included 984 subjects, recruited only nulliparous women, and the remaining trials, Wong *et al*.^11^ and Zhao *et al*.^27^, included parous parturients. The pooled results did not show a difference when these two trials were removed (OR, 0.47; 95% CI, 0.25–0.89). There was no publication bias in the trials involving CD (*P* = 0.089) and instrumental delivery (*P* = 0.747).

### Duration of Labor

**Figure 4:**
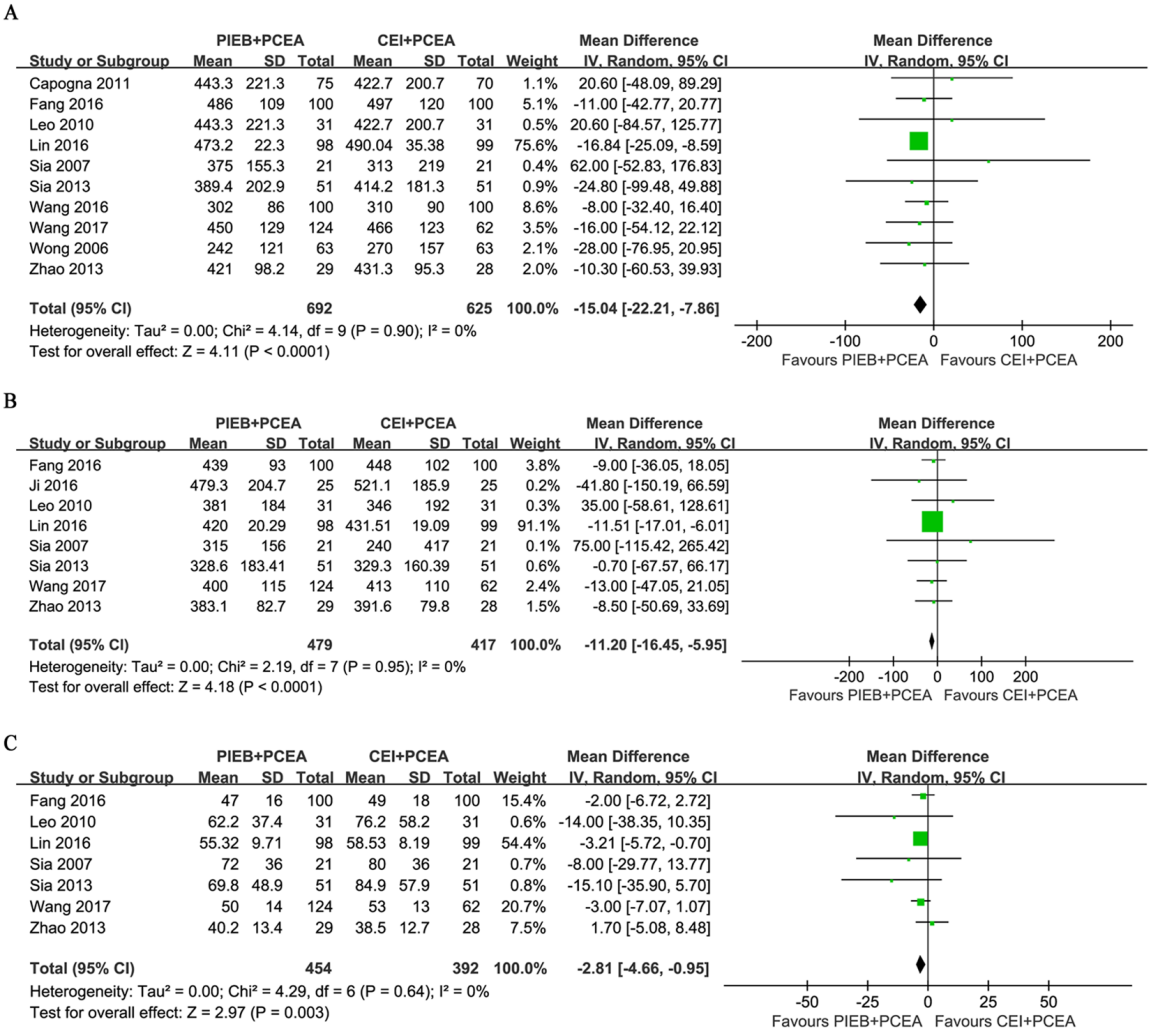
(**A**) Forest plot for the total duration (minutes) of labor. (**B**) Forest plot for the duration (minutes) of the first stage of labor. (**C**) Forest plot for the duration (minutes) of the second stage of labor. PIEB, programmed intermittent epidural boluses; CEI, continuous epidural infusion; PCEA: patient-controlled epidural analgesia; CI: confidence interval.

All trials reported the duration of labor, and ten of the studies offered information on the total duration of labor. The overall analysis showed that there was a significantly shorter total duration of labor when the PIEB + PCEA regimen was available (MD, −15.06 minutes; 95% CI, −22.16 to −7.96; *I*^2^ = 0%; *P* < 0.0001; Fig. 4A). The pooled analysis of the duration of the first stage of labor from eight reports also showed that a significantly shorter duration was observed in the PIEB + PCEA group than in the CEI + PCEA group (MD, −11.22 minutes; 95% CI, −16.44 to −6.01; *I*^2^ = 0%; *P* <0.0001; Fig. 4B). Seven trials, which included 846 subjects, reported the duration of the second stage of labor. There was a significantly shorter duration in the PIEB + PCEA group than in the CEI + PCEA group (MD, −2.83 minutes; 95% CI, −4.57 to −1.08; *I*^2^ = 0%; *P* = 0.001; Fig. 4C). There was no publication bias in the studies reporting the total duration of labor (*P* = 0.371), duration of the first stage of labor (*P* = 0.711) or duration of the second stage of labor (*P* = 0.764).

### Analgesia and Anesthesia Interventions

**Figure 5:**
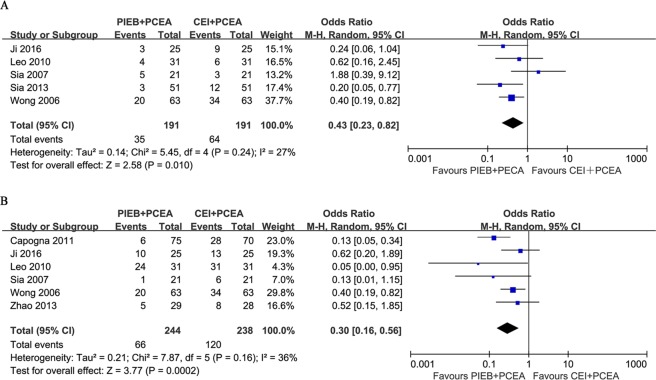
(**A**) Forest plot for the incidence of breakthrough pain. (**B**) Forest plot for the rate of using PCEA for labor analgesia. PIEB, programmed intermittent epidural boluses; CEI, continuous epidural infusion; PCEA, patient-controlled epidural analgesia; CI: confidence interval.

There was significant heterogeneity among studies regarding the timing of the VAS scoring and the assessment of average or maximal VAS values. We therefore report the results qualitatively. Nine trials estimated the efficacy of labor analgesia with a VAS at different intervals, of which 6 reports^9–11,22,24,25^ failed to observe significant differences in pain scores between the two groups at different times. Fang *et al*.^21^, Lin *et al*.^23^ and Wang^28^ reported higher VAS scores in the CEI + PCEA group than in the PIEB + PCEA group during the later stages of labor. Five trials reported the incidence of breakthrough pain^10,11,22,24,25^, which generally requires physician intervention. There was a significant reduction in the incidence of breakthrough pain in the PIEB + PCEA group compared with that in the CEI + PCEA group (OR, 0.43; 95% CI, 0.23–0.82; Fig. 5A). There was no publication bias for the studies reporting the incidence of breakthrough pain (*P* = 0.593). Six trials reported the rate of PCEA usage and showed that fewer subjects administered a bolus through the PCEA regimen in the PIEB + PCEA group, and there was a significant decrease in the rate of PCEA usage in the PIEB + PCEA group compared with that in the CEI + PCEA group (OR, 0.30; 95% CI, 0.16–0.56; Fig. 5B). There was no publication bias in the trials reporting the rate of PCEA usage (*P* = 0.976).

### Local Anesthetic Dose

**Figure 6:**
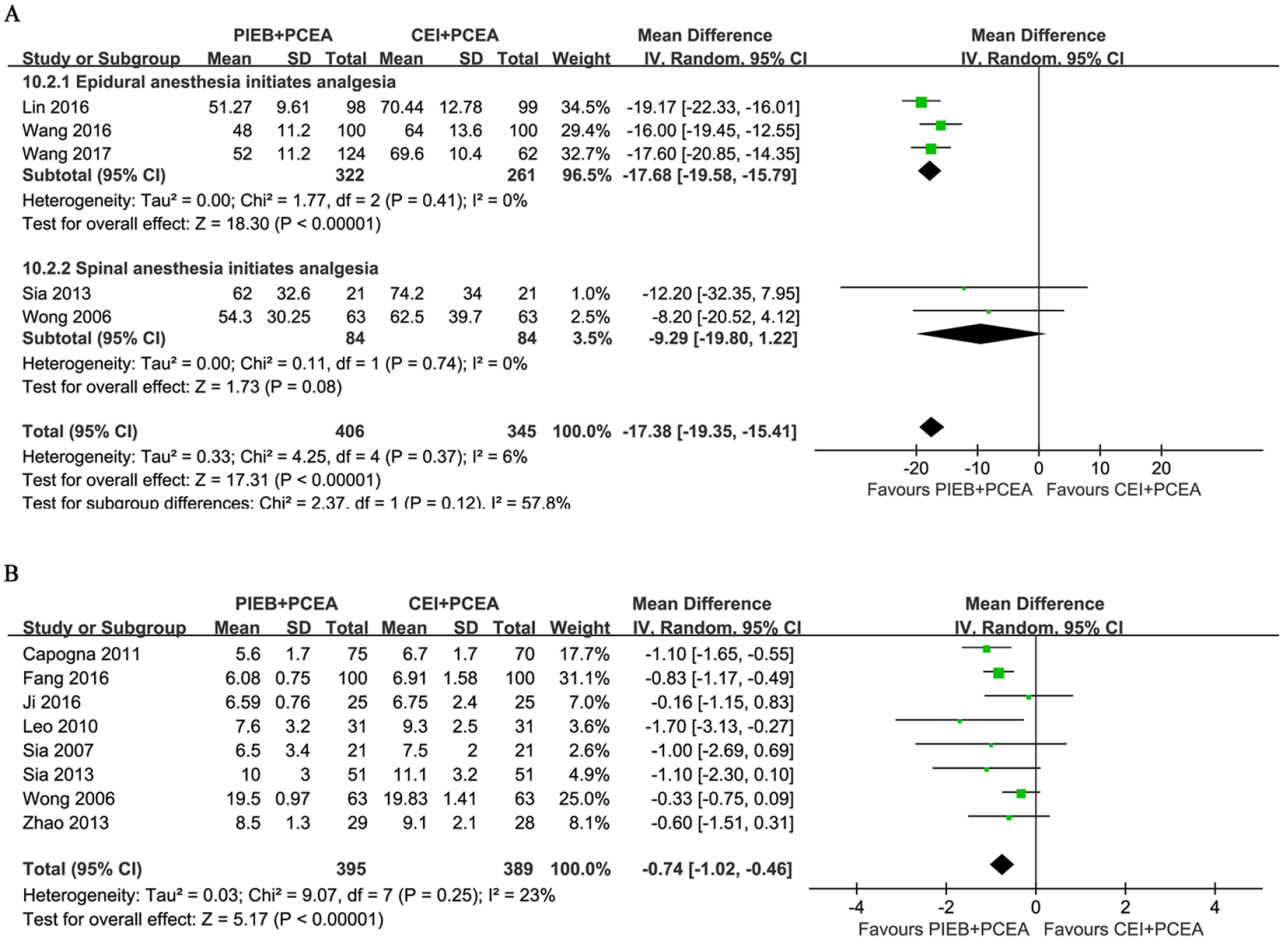
(**A**) Forest plot for total milligrams of local anesthetic (ropivacaine equivalents) consumption and the subgroup analysis for the initiating form of labor analgesia (epidural or spinal initiation). (**B**) Forest plot for milligrams per hour of local anesthetic (ropivacaine equivalents) consumption. PIEB, programmed intermittent epidural boluses; CEI, continuous epidural infusion; PCEA: patient-controlled epidural analgesia; CI: confidence interval.

The specific regimens and LA concentrations are summarized in Table 1. Five studies provided the total dose of LA administered, and a decrease in the total dose of ropivacaine was recorded for the women in the PIEB + PCEA group compared with that in women in the CEI + PCEA group; however, significant heterogeneity was observed (MD, −17.38 mg ropivacaine equivalents; 95% CI, −19.35 to −15.41; *I*^2^ = 57.8%; Fig. 6A) among these studies after pooling analysis. Therefore, a subgroup analysis was conducted, and heterogeneity was not observed when the same technique was used to initiate labor analgesia. The total dose of LA delivered per hour was extracted in eight of the published datasets^9–11,21,22,24,25,27^ including 784 participants, and the overall analysis showed that a significant decrease was observed with the PIEB + PCEA regimen compared with the CEI + PCEA regimen (MD, −0.74 mg ropivacaine equivalents per hour; 95% CI, −1.02 to −0.46; *I*^2^ = 23%; Fig. 6B). There was no publication bias in the studies reporting total (*P* = 0.452) and hourly (*P* = 0.129) LA delivered.

### Maternal Satisfaction

**Figure 7:**
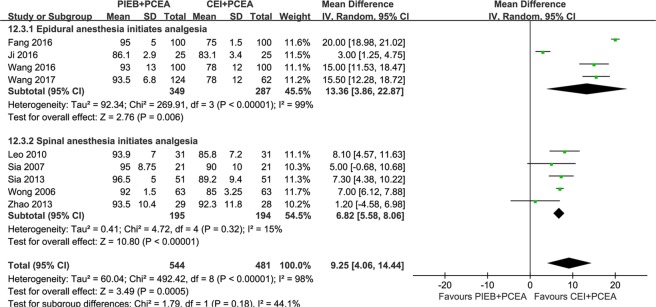
Forest plot for maternal satisfaction (visual analog scale [VAS] 0–100 mm; 0 represents very dissatisfied, 100 represents extremely satisfied) and the subgroup analysis for the initial form of labor analgesia (epidural or spinal initiation). PIEB, programmed intermittent epidural boluses; CEI, continuous epidural infusion; PCEA: patient-controlled epidural analgesia; CI: confidence interval.

Nine trials reported overall maternal satisfaction with labor analgesia, and most studies used a verbal rating scale (VRS) in which 0 represented very dissatisfied and 10 or 100 represented extremely satisfied. Only one trial reported maternal satisfaction with a visual analog scale (VAS) score^11^. All trials, except for Sia *et al*.^25^ and Zhao *et al*.^27^, reported higher maternal satisfaction scores in the PIEB + PCEA group than in the CEI + PCEA group; the overall analysis showed that participants had a significantly higher maternal satisfaction score in the PIEB + PCA group than in the CEI + PCEA group (MD, 9.25; 95% CI, 4.06 to 14.44; Fig. 7), but significant heterogeneity was observed (*I*^2^ = 98%) among these studies. A subgroup analysis was thus conducted. The subgroup of subarachnoid anesthesia initiation showed a significantly lower heterogeneity (*I*^2^ = 15%) among the trials, while a higher heterogeneity was still observed in the subgroup using epidural technique initiation (*I*^2^ = 99%). There was no publication bias in the trials reporting maternal satisfaction (*P* = 0.608).

### Additional Outcomes

The incidence of motor block, assessed with the Breen-modified Bromage score^9,29^ <6 or a traditional Bromage score^11,21,22,24–26,28^ >1, occurring at least once during labor (Table 2) was significantly increased in the CEI group compared with the PIEB group, but significant heterogeneity was observed (*I*^2^ = 54%). A sensitivity analysis suggested that Capogna *et al*.^9^ appeared to increase the heterogeneity and used the Breen-modified Bromage score^29^, which is different than the Bromage score used by other trials; after removing this trial, the overall results showed no significant difference between the two regimens.

**Table 2:** Additional Outcomes.

Outcome	Studies	Number of events/total in PIEB + PCEA group	Number of events/total in CEI + PCEA group	Odds ratio (95% confidence interval)	*I*^*2*^ (%)	*P-*Value
Motor blockade	Capogna *et al*.^9^ Fang *et al*.^21^ Ji *et al*.^22^ Sia *et al*.^25^ Sia *et al*.^24^ Wang *et al*.^26^ Wang *et al*.^28^ Wong *et al*.^11^	8/435 (1%)	34/430 (6.7%)	0.18 [0.09, 0.38]	54	<0.0001
Hypotension	Fang *et al*.^21^ Ji *et al*.^22^ Leo *et al*.^10^ Sia *et al*.^25^ Sia *et al*.^24^ Wang *et al*.^26^ Zhao *et al*.^27^	4/357 (1.1%)	8/356 (2.5%)	0.53 [0.17, 1.64]	0	0.27
Nausea/vomiting	Fang *et al*.^21^ Ji *et al*.^22^ Leo *et al*.^10^ Sia *et al*.^25^ Sia *et al*.^24^ Wang *et al*.^26^	6/328 (1.8%)	4/328 (1.2%)	1.47 [0.43, 5.03]	0	0.54
Pruritus	Fang *et al*.^21^ Leo *et al*.^10^ Sia *et al*.^25^ Sia *et al*.^24^ Wang *et al*.^26^	69/303 (22.8%)	65/303 (21.5%)	1.13 [0.70, 1.84]	0	0.62

PIEB, programmed intermittent epidural boluses; CEI, continuous epidural infusion; PCEA: patient-controlled epidural analgesia.

Six trials reported 1-minute Apgar scores^21–23,26,27^. There was no difference between the 2 groups in any trials. Eight trials reported the 5-minute Apgar scores, of which five trials gave the concrete scores^10,23,25–27^. There were no significant differences observed between the two regimens across all trials. Fang *et al*.^21^ and Wang *et al*.^28^ reported that the 5-minute Apgar score for all subjects exceeded 7. Sia *et al*.^25^ reported that 81% (17/21) of the subjects in the PIEB + PCEA group and 90% (19/21) of subjects in the CEI + PCEA group surpassed scores of seven. Significant differences were not observed between the two groups.

Other maternal side effects (nausea/vomiting, pruritus, hypotension) are listed in Table 2. Significant differences were not found between the two regimens. Neonatal adverse effects (fetal bradycardia) were not reported in these trials except in Leo *et al*.^10^ and Sia *et al*.^24^, and there was no difference between the two groups.

## Discussion

The important findings of this systematic review and meta-analysis study show that PIEB in conjunction with the PCEA regimen is of greater benefit to the parturient and fetus. In these healthy women requesting labor epidural analgesia, PIEB as the background infusion of the PCEA, compared with CEI plus PCEA, significantly reduced the risk of instrumental delivery, improved labor pain and pain relief, reduced LA consumption, resulted in higher maternal satisfaction, and statistically shortened the labor duration, but there were no differences with regard to the incidence of adverse events (motor block, Apgar score, etc.).

PCEA allowed the parturient to self-administer a bolus and thereby reduce the time between the onset of pain and the administration of additional analgesia^8^. CEI with or without PCEA boluses has been regarded as standard labor epidural analgesic regimens in many institutions in North America and Europe^16^. However, a higher risk of instrumental delivery exists when CEI was added to the PCEA regimen than for the PCEA-only regimen^8^. Multiple studies have found that the rate of instrumental delivery was decreased when PIEB was added to PCEA compared with that for CEI + PCEA^9,28,30^, but the 2013 systematic review by George *et al*.^16^ did not show a significant difference in the combined results. Our study showed that PIEB reduced the risk of instrumental delivery compared with CEI when pooling the data reported in the recent studies that conducted a comparison of PIEB and CEI being added to the PCEA regimen.

The incidence of instrumental delivery is associated with pelvic muscle tone, motor blockade and the ability to “bear down” during the second stage of labor^31^. A lower incidence of motor blockade was observed with the PIEB + PCEA regimen than with the CEI + PCEA regimen. Motor blockade may influence the duration of labor, and the use of PCEA for maintenance of labor analgesia may have a noteworthy effect on the duration of the first stage and the total duration of labor. George *et al*.^16^ showed that the duration of the second stage was as much as 22 minutes shorter with IEB alone than with the CEI with or without PCEA, and when IEB added to PCEA was used for maintenance of labor, the duration of the first stage was longer (20 minutes). The total duration of labor was reduced (−4 minutes) compared with that for CEI, and thus, they speculated that IEB may enter the realm of clinical significance and positively affect labor progression. Our pooled results showed a statistically significant difference (−15, −11 and −3 minutes) in the length of total, first and second stage of labor, but such differences are hardly clinically significant.

In fact, patients’ preferences about labor are focused on both pain relief and labor duration, and patients preferred lower intensity, longer duration to higher intensity, short duration labor pain; thus, direct management of labor pain is needed to meet patient expectations^32,33^. A superior efficacy of labor analgesia for parturients with the PIEB + PCEA regimen was presented in our study, and there was a significant decrease in the incidence of breakthrough pain, which is a transitory exacerbation of pain that occurs once previously used labor analgesia becomes ineffective and often requires supplemental epidural medications using a PCEA regimen or manual bolus^34–37^. A lower rate of PCEA usage was also observed, and the time to first PCEA usage or first breakthrough pain was postponed in most included studies^10,11,22,24,25,27^. The relief of labor analgesia is partially contingent upon a greater LA consumption^38^. Our pooled results showed that the total or hourly dose of LA was markedly decreased in the PIEB + PCEA group compared with that in the CEI + PCEA group. Lower LA usage to achieve optimal analgesia was indeed disputed between the PIEB or PCEA regimen maintaining labor analgesia, likely due to lack of a standardized useful tool to assess the degree of pain. The VAS is one of the most commonly used methods^39^, but when an acceptable VAS pain score for parturients is a consistently controlled score of less than 3^40^, a significant difference could not occur easily; once the scores were greater than 3, the parturients or anesthetists would take measures to relieve the pain, therefore a difference would not be observed.

The main goal of labor analgesia is to accomplish an ideal and desired level of pain relief and greater satisfaction of care provided to the patients. Maternal satisfaction, a multidimensional measure, is a common evaluation method that involves personal expectations, labor pain, perception of emotional control, communication skills of her caregiver and maternal involvement in decision making^40–43^. The meta-analysis and review by George *et al*.^16^ stated that PIEB with or without PCEA slightly improved maternal satisfaction. Our study showed that PIEB + PCEA resulted in significantly higher maternal satisfaction scores than CEI + PCEA, but the aggregated studies had significant heterogeneity (*I*^2^ = 98%). The use of a different neuraxial analgesia technique to initiate labor analgesia might have influenced maternal satisfaction. Our subgroup analysis suggested that heterogeneity was especially apparent when an epidural technique was used to initiate labor analgesia, likely due to the analgesia efficacy of the epidural technique itself being influenced by many factors (concentration, volume, composition of medicine, etc.). In fact, women’s self-diagnosis of the onset of labor and their perception of their labor duration when meeting their midwives has some impact on their admission to the labor ward and the timing of epidural analgesia^32,44^; thus, it is difficult to accurately measure maternal satisfaction at standard intervals or the same times with the standard assessment tools, all of which could cause heterogeneity, so the pooled results have to be interpreted with caution.

There are several limitations to this study. First, none of the included studies reported all of the relevant outcomes, thus limiting the statistical power of this systematic review. Second, labor analgesia was not evaluated in the same way; some trials recorded VAS scores during the overall labor progression at different time points, while several studies used the incidence of breakthrough pain to indirectly reflect labor analgesia. Third, most trials nearly consistently reported maternal satisfaction to evaluate the relief of labor pain, but significant heterogeneity was observed among studies. Fourth, only two trials recruited parous subjects; the remaining studies included uncomplicated nulliparous women, which likely limits the conclusions to be extrapolated to women presenting with multiple gestations and parous women. Fifth, despite only these trials directly comparing the PIEB + PCEA regimen with the CEI + PCEA regimen were included in this systematic review, significant clinical heterogeneity with respect to labor analgesia initiation, LA concentration or volume and drug delivery regimens still existed, which may affect maternal and obstetric outcomes^28,45^.

In conclusion, the PIEB + PCEA regimen, compared with the CEI + PCEA regimen, showed greater benefit for decreasing the risk of instrumental delivery, relieving labor pain, decreasing LA consumption, and improving maternal satisfaction, but future prospective and adequately powered studies should be conducted to confirm earlier findings and optimize the PIEB settings with respect to LA concentration, volume boluses, time intervals, etc. Given that women’s own perceptions and expectations regarding the onset and process of labor affect the evaluation of satisfaction, educational strategies for the implementation of these techniques should be taken to guide pump programming.

## Supplementary information

PCEA PRISMA-Supplemental Appendix S1

Supplemental Appendix S2
