# Supplementary material for: A Systematic Review and Meta-Analysis Comparing Programmed Intermittent Bolus and Continuous Infusion as the Background Infusion for Parturient-Controlled Epidural Analgesia
Source: Sci Rep. 2019 Feb 22;9:2583. doi: 10.1038/s41598-019-39248-5 (PMC6384894; doi:10.1038/s41598-019-39248-5)
Supplement: Supplementary file 2 — Supplemental Appendix S2 [file 41598_2019_39248_MOESM2_ESM.doc]

**Supplemental Appendix S2**

**A Systematic Review and Meta-Analysis Comparing Programmed Intermittent Bolus and Continuous Infusion as the Background Infusion for Parturient-Controlled Epidural Analgesia**

**Jiqian Xu^1,2,3^,** **^†^, Jie Zhou^4, †^, Hairong Xiao^1,3^**, **Shangwen Pan^3^, Jie Liu^2^**, **You Shang^3^,** **Shanglong Yao^1,3^***

**Search strategies (S1 Fig. Search strategy of this study.)**

Ⅰ. PubMed (Medline) search strategy (February 15, 2018)

((((Pregnancy) AND epidural analgesia) AND (((programmed) OR automated) OR intermittent))) OR (((((((Pregnancies[Title/Abstract]) OR Gestation[Title/Abstract])) OR "Pregnancy"[Mesh])) AND ((Epidural Analgesia[Title/Abstract]) OR "Analgesia, Epidural"[Mesh])) AND (((intermittent [Text Word]) OR automated[Text Word]) OR programmed[Text Word]))

Ⅱ. Embase (February 15, 2018)

1 'epidural analgesia'.mp. [mp=ti, ab, tx, ct, sh, hw, tn, ot, dm, mf, dv, kw, fx, bt, nm, kf, px, rx, an, eu, pm, ui, ds, on, sy]

2 'Epidural Analgesia'.ab,ti.

3 1 or 2

4 'Pregnancy'.mp. [mp=ti, ab, tx, ct, sh, hw, tn, ot, dm, mf, dv, kw, fx, bt, nm, kf, px, rx, an, eu, pm, ui, ds, on, sy]

5 'Pregnancies'.ab,ti.

6 'Gestation'.ab,ti.

7 4 or 5 or 6

8 'intermittent'.ab,ti.

9 'automated'.ab,ti.

10 'programmed'.ab,ti.

11 8 or 9 or 10

12 3 and 7 and 11

Ⅲ. Cochrane Central Register of Controlled Trials (February 15, 2018)

#1 MeSH descriptor: [Pregnancy] explode all trees

#2 Pregnancies:ti,ab,kw or Gestation:ti,ab,kw (Word variations have been searched)

#3 Intermittent:ti,ab,kw or Automated:ti,ab,kw or Programmed:ti,ab,kw (Word variations have been searched)

#4 MeSH descriptor: [Analgesia, Epidural] explode all trees

#5 epidural analgesia:ti,ab,kw (Word variations have been searched)

#6 #1 or #2

#7 #4 or #5

#8 #3 and #6 and #7

Ⅳ. Web of science (February 15, 2018)

#1 TS=(Pregnancy OR Pregnancies OR Gestation)

#2 TS=(Epidural Analgesia OR epidural analgesia)

#3 TS=(Intermittent OR Automated OR Programmed)

#4 #1 and #2 and #3
